# Epidermal Growth Factor Receptors and Breast Cancer

**Published:** 1989-05

**Authors:** J. R. Farndon, S. Nicholson, J. R. C. Sainsbury, A. L. Harris

**Affiliations:** Professor of Surgery, University of Bristol; Registrar in Surgery, Northern Regional Rotation Scheme; Consultant Surgeon, Huddersfield Royal Infirmary; Professor of Clinical Oncology, University of Oxford


### Epidermal Growth Factor Receptors and Breast Cancer

**J. R. Farndon**
Professor of Surgery, University of Bristol
**S. Nicholson**
Registrar in Surgery, Northern Regional Rotation Scheme
**J. R. C. Sainsbury**
Consultant Surgeon, Huddersfield Royal Infirmary
**A. L.Harris**
Professor of Clinical Oncology, University of Oxford

Growth factors are polypeptides that stimulate cell proliferation through binding to specific high-affinity cell membrane receptors. Plasma contains small quantities of growth factors. Platelets contain large amounts and it is thought that these are delivered to sites of injury where they may influence wound healing and repair.

Growth factors are present in a variety of other tissues and have differing cell specificities. Much interest has recently centred on these agents because of their possible involvement in malignant processes. Oncogenes and growth factors are thought to be closely related or linked, for example, the v-erb-B oncogene codes for the Epidermal Growth Factor receptor (EGFr). Growth factors have been shown to increase the transcription of certain proto-oncogenes, products of which may in turn regulate the transcription of other genes necessary for stimulation of cell proliferation.

Epidermal growth factor (EGF) is peptide of molecular weight 6.045 kDa which stimulates cell proliferation in vitro and in vivo. It was first isolated from mouse submaxillary glands by Cohen in 1962. If EGF is injected into new born mice it induces precocious eyelid opening and incisor eruption (1). In 1975 Gregory showed that human EGF was equivalent to a previously characterized substance urogastrone, a hormone capable of inhibiting gastric acid secretion. Mouse EGF and urogastrone share a 70% homology, are distinct in radiommunoassay but react similarly in radioligand and growth stimulation experiments.

The biological effects of EGF are mediated through high-affinity binding to specific membrane receptors which are integral 170 kDa membrane proteins (2). The degree of homology to the v-erb-B tranforming protein of the avian erythroblastosis virus suggests that the EGF receptor has a cytoplasmic domain of approximately 60 kDa, a short transmembrane sequence and a large 100 kDa external domain which is present in a truncated form in the v-erb-B protein. The external domain shares the binding site for EGF and the cytoplasmic domain contains intrinsic EGF activated tyrosine kinase activity (2).

We were first interested to examine breast cancers for the presence of EGFr following the report of the effects of EGF on certain mammary cell lines in vitro (3). Our initial studies published in 1985 demonstrated that 1/5 of all primary breast tumours expressed EFGr and a much higher proportion of metastatic disease appears to exhibit the receptor (4). An inverse relationship was found between EGFr and the oestrogen receptor status (ER) of the tumour. If a tumour expressed EGFr it was unlikely to be ER positive and vice versa. It appeared that the EGFr positive tumours were of poor differentiation and had characteristics associated with a poor prognosis. We went on to demonstrate that the EGFr could be detected by immunocytochemical techniques with similar results. The underlying conclusion was that EGFr were associated with metastatic potential and that the growth of a proportion of poor prognosis ER-ve tumours may be regulated by peptide growth factors interacting with the EGFr (5). Others confirmed our findings (6). It was shown that as many as 42% of breast cancers might be EGFr positive. The same inverse relationship with the ER was demonstrated and nearly all metastatic deposits were found to be EGFr positive. Battaglia et al (6) suggested that a poor prognosis group of tumours existed which were stimulated by EGF or EGF-like substances rather than steroid hormones and that this group of tumours would be unresponsive to endocrine therapy.

### ASSAY METHODS AND CLINICAL APPLICABILITY

We have gone on to demonstrate that a two point assay using 1 nM labelled EGF correlated well with values obtained for high-affinity binding sites from full Scratchard analysis (7). Analysing a total of 246 breast cancers confirmed the presence of a strong inverse relationship between EGFr and ER. Follow-up data from 135 patients revealed a strong inverse relationship between EGFr greater than 10 fmol/mg of membrane protein and ER status and a positive correlation with early recurrence and death. A reproducible two point assay system can be used, a cut-off point of 10 fmol/mg of membrane protein allows clinically useful data to be produced. Unfortunately the presence of EGFr demonstrates a poor prognosis, endocrine unresponsive tumour.

### EGFr AND HISTOLOGICAL SUB-TYPES OF BREAST

Careful histological characterization of consecutive series of 264 surgically resected malignant breast tumours was defined and their ER and EGFr determined. The tumour size and lymph node involvement was noted. Those non-ductal tumours containing EGFr compared with 34% of ductal carcinomas (Table).

**Relationship of EGF receptor and oestrogen receptor for ductal and lobular cancers**

| | | *EGFr Positive* | *Negative* | |
|---|---|---|---|---|
| Ductal ER | positive | 10 | 84 | |
| | negative | 71 | 74 | 239 |
| | | $X^2=35.7$; $P<0.001$ | | |
| Lobular ER | positive | 0 | 6 | |
| | negative | 3 | 5 | |
| | | | | 14 |
| Histology | | | | |
| Ductal | 81 | 158 | | |
| Lobular | 3 | 11 | | |
| | | $X^2=0.44$; $P=0.5$ | | 253 |

EGFr appeared to be associated with an increased risk of early recurrence and death whatever the histological sub-type of breast cancer (8).

### EGFr, RECURRENT BREAST CANCER AND ENDOCRINE RESPONSE

By measuring the EGFr and ER receptor status of tumours after surgery it is possible that these biological markers provide information about the likely response to endocrine therapy at relapse. After a median follow-up of 24 months 99 patients of 221 with primary operable breast tumours had relapse. Seventy-two of these received tamoxifen alone as first line treatment for recurrence (median age 56 years, range 32–77 years). Twenty patients (28%) showed a response to this therapy and 52 (72%) did not. Of 32 ER positive tumours, 12 (37.5%) showed an objective response to tamoxifen compared with only two of 40 (5%) ER negative tumours (P=<0.005). Of 35 EGFr positive tumours, three (8.5%) achieved an objective response compared with 11 of 37 (30%) EGFr negative tumours (P=<0.05). Only one of 28 EGFr positive, ER negative tumours achieved an objective response. If we include patients whose disease remains stable for more than six months with the responders, however, EGFr status was a better predictor of response to tamoxifen; 15 of 37 EGFr negative patients and five of 35 EGFr positive patients responded (P=<0.01), compared with 13 of 32 ER positive and seven of 40 ER negative patients (not significant). We emphasize, therefore, that EGFr expression is a highly significant marker of poor prognosis in patients with breast cancer. It appears to be as good a predictor as ER for objective response and better for overall response to endocrine therapy on relapse (9).

### EGFr IN ELDERLY PATIENTS WITH BREAST CANCER

Current clinical practice commonly dictates that elderly patients (over 65 years old) with breast cancer will respond primarily to endocrine therapy in a high proportion of instances (perhaps more than 60%).

Clinical observation suggested that the proportion of patients unresponsive to primary endocrine therapy often did as badly as pre-menopausal patients with the development of visceral or skeletal metastases progressing to death. We, therefore, studied the predictive value of EGFr in response to primary endocrine therapy in elderly females.

Sixty-one elderly women (median age 77 years) received primary endocrine therapy for operable breast cancer. Eleven patients (18%) had complete and 24 (39%) partial regression, 12 (20%) had stable disease for a minimum period of six months and 14 (23%) showed no response. Salvage surgery was undertaken in the 14 with no response and eight of nine with progressive disease following initial response. Tumour samples were available, therefore, from patients with relapsed disease only. Assays for EGFr and ER were performed on material from 20 of 22 such patients. Ten of these 20 tumours were EGFr positive (>10 fmols/mg) and nine of 13 patients progressing within six months had EGFr positive tumours. No such association was seen with ER status. EGFr status was associated with early recurrence after surgery and death in the endocrine failed group (P=<0.005 and P=<0.05 respectively).

In a control population of 33 patients (median age 72 years) treated by surgery alone only six patients were EGFr positive. In this group early relapse was predicted by EGFr status but not by ER status (median disease free survival for EGFr positive patients 15 months and for EGFr negative patients 40 months, P=<0.01, log rank test).

There was a significantly higher proportion of EGFr positive tumours in the endocrine failure group compared with the control population and EGFr status therefore is a marker for rapid early progression on primary endocrine therapy. The development of non-excisional methods of EGFr analysis would allow better directed therapeutic decisions in this elderly group of patients (10).

### CONCLUSION

The receptor to epidermal growth factor is clearly a significant biological marker in breast cancer. Its measurement might best direct methods of therapy in all age groups. By and large it identifies the aggressive tumour associated with poor prognosis. The identification of patients with such tumours might categorize them to receive more aggressive therapies such as chemotherapy. Methods of blocking the EGFr might have therapeutic possibilities. The attachment of chemotherapeutic agents by covalent bonds to EGF ligand might allow delivery of chemotherapeutic agents more precisely into tumour mass and allow better cytotoxic kill.

### ACKNOWLEDGEMENT

We are grateful to the North of England Cancer Research Campaign for support of this research work.

### REFERENCES

1 COHEN, S. (1962) Isolation of a mouse submaxillary gland protein accelerating incisor eruption and eyelid opening in the newborn animal. *J. Biol. Chem.* **237**, 1555–1562.

2 COHEN, S., USHIRO, H., STOSCHECK, C., CHINKERS, M. (1982) A native 170,000 epidermal growth factor receptor-kinase complex from shed membrane vesicles. *J. Biol. Chem.* **313**, 491–492.

3 IMAI, Y., LEUNG, C. K., FRIESON, H. G. *et al.* (1982) Epidermal growth factor receptors and effect of epidermal growth factor on growth of human breast cancer cells in long term tissue culture. *Cancer Res.* **42**, 4394–4398.

4 SAINSBURY, J. R. C., FARNDON, J. R., HARRIS, A. L., SHERBET, G. V. (1985) Epidermal growth factor receptors on human breast cancers. *Br. J. Surg.* **72**, 186–188.

5 SAINSBURY, J. R. C., FARNDON, J. R., HARRIS, A. L., SHERBET, G. V. (1985) Epidermal growth factor receptors and oestrogen receptors in human breast cancer. *The Lancet*, Feb, 364–366.

6 BATTAGLIA, F., POLIZZI, G., SCAMBIA, G., ROSSI, S., PANICI, P. B., IACOBELLI, S. *et al.* (1988) Receptors for epidermal growth factor and steroid hormones in human breast cancer. *Oncology* **45**, 424–427.

7 NICHOLSON, S., SAINSBURY, J. R. C., NEEDHAM, G. K., CHAMBERS, P., FARNDON, J. R., HARRIS, A. L. (1988) Quantitative assays of epidermal growth factor receptor in human breast cancer: cut-off points of clinical relevance. *Int. J. Cancer* **42**, 36–41.

8 SAINSBURY, J. R. C., NICHOLSON, S., ANGUS, B., FARNDON, J. R., MALCOLM, A. J., HARRIS, A. L. (1988) Epidermal growth factor receptor status of histological sub-types of breast cancer. *Br. J. Cancer* **58**, 458–460.

9 NICHOLSON, S., SAINSBURY, J. R. C., HALCROW, P., CHAMBERS, P., FARNDON, J. R., HARRIS A. L. (1989) Expression of epidermal growth factor receptors associated with lack of response to endocrine therapy in recurrent breast cancer. *The Lancet* Jan 182–184.

10 NICHOLSON, S., HALCROW, P., SAINSBURY, J. R. C., ANGUS, B., CHAMBERS, P., FARNDON, J. R., HARRIS, A. L. (1988) Epidermal growth factor receptor (EGFr) status associated with failure of primary endocrine therapy in elderly postmenopausal patients with breast cancer. *Br. J. Cancer* **58**, 810–814.

